# Assessing Wound Healing in Vivo Using a Dual-Function Phosphorescent Probe Sensitive to Tissue Oxygenation and Regenerating Collagen

**DOI:** 10.1021/acsami.4c15069

**Published:** 2024-12-27

**Authors:** Xiaoyan Wang, Zhiming Zhang, Xuhao Ye, Liping Chen, Weiming Zheng, Ning Zeng, Zhouji Shen, Fei Guo, Igor O. Koshevoy, Kristina S. Kisel, Pi-Tai Chou, Tzu-Ming Liu

**Affiliations:** † Institute of Translational Medicine, Faculty of Health Sciences & Ministry of Education Frontiers Science Center for Precision Oncology, University of Macau, Taipa 999078, Macau, China; ‡ Department of Pediatric Surgery, Guangzhou Institute of Pediatrics, Guangdong Provincial Key Laboratory of Research in Structural Birth Defect Disease, Guangzhou Women and Children’s Medical Center, Guangzhou Medical University, Guangzhou 510623, Guangdong, China; § First Department of Hepatobiliary Surgery, Zhujiang Hospital, Southern Medical University, Guangzhou 510280, China; ∥ Guangdong Provincial Clinical and Engineering Center of Digital Medicine, Guangzhou 510280, China; ⊥ Ningbo Medical Center LiHuiLi Hospital, The Affiliated LiHuiLi Hospital of Ningbo University, Ningbo, Zhejiang 315040, China; ■ Ningbo Institute of Innovation for Combined Medicine and Engineering (NIIME), The Affiliated Lihuili Hospital of Ningbo University, Ningbo, Zhejiang 315040, China; ○ Department of Chemistry, University of Eastern Finland, FI-70211 Joensuu, Finland; ∇ Department of Chemistry, National Taiwan University, Taipei 10617, Taiwan

**Keywords:** Phosphorescence probe, Phosphorescence lifetime imaging, Wound healing, Collagen regeneration, Wound oxygenation

## Abstract

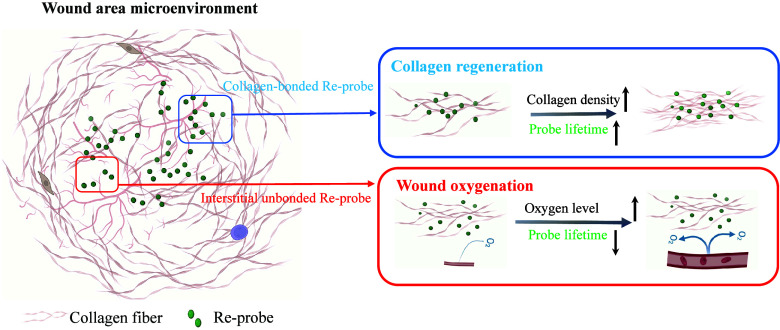

Levels of tissue oxygenation and collagen regeneration are critical indicators in the early evaluation of wound healing. Traditionally, these factors have been assessed using separate instruments and different methodologies. Here, we adopt the spatially averaged phosphorescence lifetime approach using Re^I^-diimine complexes (Re^I^-probe) to enable simultaneous quantification of these two critical factors in healing wounds. The topically applied, biocompatible Re^I^-probe penetrates wound tissue effectively and selectively binds to collagen fibers. During collagen regeneration, the phosphorescence lifetimes of the collagen-bound probe significantly extend from an initial range of 4.5–6.5 μs on day 0 to 5.5–8.5 μs by day 7. Concurrently, unbound probes in the tissue interstitial spaces exhibit a phosphorescence lifetime of 4.5–5.2 μs, revealing the oxygenation states. Using phosphorescence lifetime imaging microscopy (PLIM) and a frequency domain phosphorescence lifetime measurement (FD-PLM) system, we validated the dual-functionality of this Re^I^-probe in differentiating healing stages in chronic wounds. With its noninvasive, quantitative measurement capabilities for cutaneous wounds, this Re^I^-probe-based approach offers promising potential for early wound healing diagnosis.

## Introduction

Nonhealing chronic wounds pose a significant global public health challenge. Despite advancements in materials engineering that support tissue regeneration,^1,2^ the complex microenvironment of chronic wounds—including biofilm formation,^3^ chronic inflammation, and impaired blood perfusion—continues to impede treatment progress. Timely assessment and early intervention are essential, as they can increase cure rates by preventing wound deterioration and complications,^4^ reduce healthcare costs by shortening hospital stays and minimizing resource use,^5^ and improve patient quality of life by alleviating pain and promoting faster recovery. Extensive research has explored key biological parameters of wounds such as pH value, oxygen level, collagen remodeling, and inflammation.^6−8^ Oxygenation status, indicative of neovascularization and blood perfusion in a healing wound, directly impacts the quality of new tissue formation. Oxygen levels are frequently measured to guide treatment plans and critical decisions, including potential amputation.^9^ Regenerated collagen, essential for supporting resident cells, plays a crucial role in maintaining normal tissue function. Its dynamic deposition, maturation, and remodeling in the extracellular matrix are fundamental to successful wound healing.^8^ Disruptions in these processes can lead to a chronic, nonhealing state. From a diagnostic perspective, visualizing and quantifying dynamic changes in tissue oxygenation and collagen regeneration in wounds is essential.

Traditionally, tissue oxygen levels have been measured using invasive O_2_ microelectrodes, which lack spatial information on O_2_ distribution *in vivo*.^10^ With advances in molecular imaging techniques, the O_2_ concentration in wounds can now be mapped with minimal invasiveness. Optical imaging of hypoxia-responsive probes, in particular, has demonstrated significant advantages due to its high sensitivity, noninvasiveness, subcellular resolution, and operational ease.^11−14^ By leveraging oxygen quenching of phosphorescence, transition-metal probes have been widely used to quantify oxygen concentration through their phosphorescent lifetimes or intensity decay during wound repair and healing.^15,16^ However, as most phosphorescent probes are hydrophobic and require encapsulation in carriers, achieving biocompatibility and deep penetration in neotissues remains challenging.^17^ Collagen regeneration is often observed or analyzed through various techniques including biochemical assays, histological staining, and polarized light microscopy. In virtual optical biopsy, the noncentrosymmetric structure of collagen allows for its label-free visualization within wounds using second harmonic generation (SHG) microscopy.^18,19^ This advanced method is frequently employed to discern changes in collagen fiber orientation and textures for disease diagnosis.^20,21^ However, enhancing the sensitivity of collagen detection requires greater optical energy or higher numerical aperture objectives, which limit the scanning speed across large wound areas. Currently, no single technology adequately addresses both clinical needs, making simultaneous observation and measurement of tissue oxygen and collagen reconstruction complex and cumbersome. This challenge highlights the need for a streamlined solution to enable comprehensive wound assessment in clinical routines.

One strategy for simultaneous imaging of two or more functional parameters is to integrate multiple indicators within a single carrier.^22^ For instance, concurrent measurement of oxygen and carbon dioxide at the same location has been achieved to elucidate processes differentiating oxidative respiration and photosynthesis.^23^ Other indicators, such as pH/O_2_ and pH/Temperature,^16,24,25^ have also been developed for dual-parameter measurements. However, to date, no functional probes have successfully enabled the simultaneous imaging of oxygenation and collagen in live biological tissues.

In this work, we demonstrate a dual-function phosphorescence probe based on the Re^I^-diimine luminescent complex (Re^I^-probe),^26^ capable of achieving simultaneous and accurate assessment of both wound oxygenation status and collagen regeneration. This water-soluble probe penetrates blood vessels easily and diffuses effectively into dermis tissue, where it binds selectively to collagen. Upon collagen binding, the range of oxygen-dependent phosphorescent lifetime in the Re^I^-probe significantly increased from 1.5–4 μs to 5–7.5 μs. By exploiting this unique collagen-induced lifetime switching property, we achieved semiquantitative measurement of collagen deposition density via spatially averaged phosphorescence lifetime. Concurrently, unbound probes within tissue interstitial spaces exhibit a lifetime range of 4.5–5.2 μs, revealing the tissue oxygenation status. Integrated with either phosphorescence lifetime imaging microscopy (PLIM) or frequency domain phosphorescence lifetime measurement (FD-PLM) system, we were able to noninvasively track the *in situ* phosphorescence lifetime change at different stages and locations of wound healing. Given its performance, reliability, and noninvasive advantages, this integrated system should facilitate chronic wound diagnosis and guide clinic treatment strategies.

## Results and Discussion

### Permeated Re^I^-Probes in Wounds Can Sensitively Reveal Collagen Morphology with Biocompatibility

**Figure 1:**
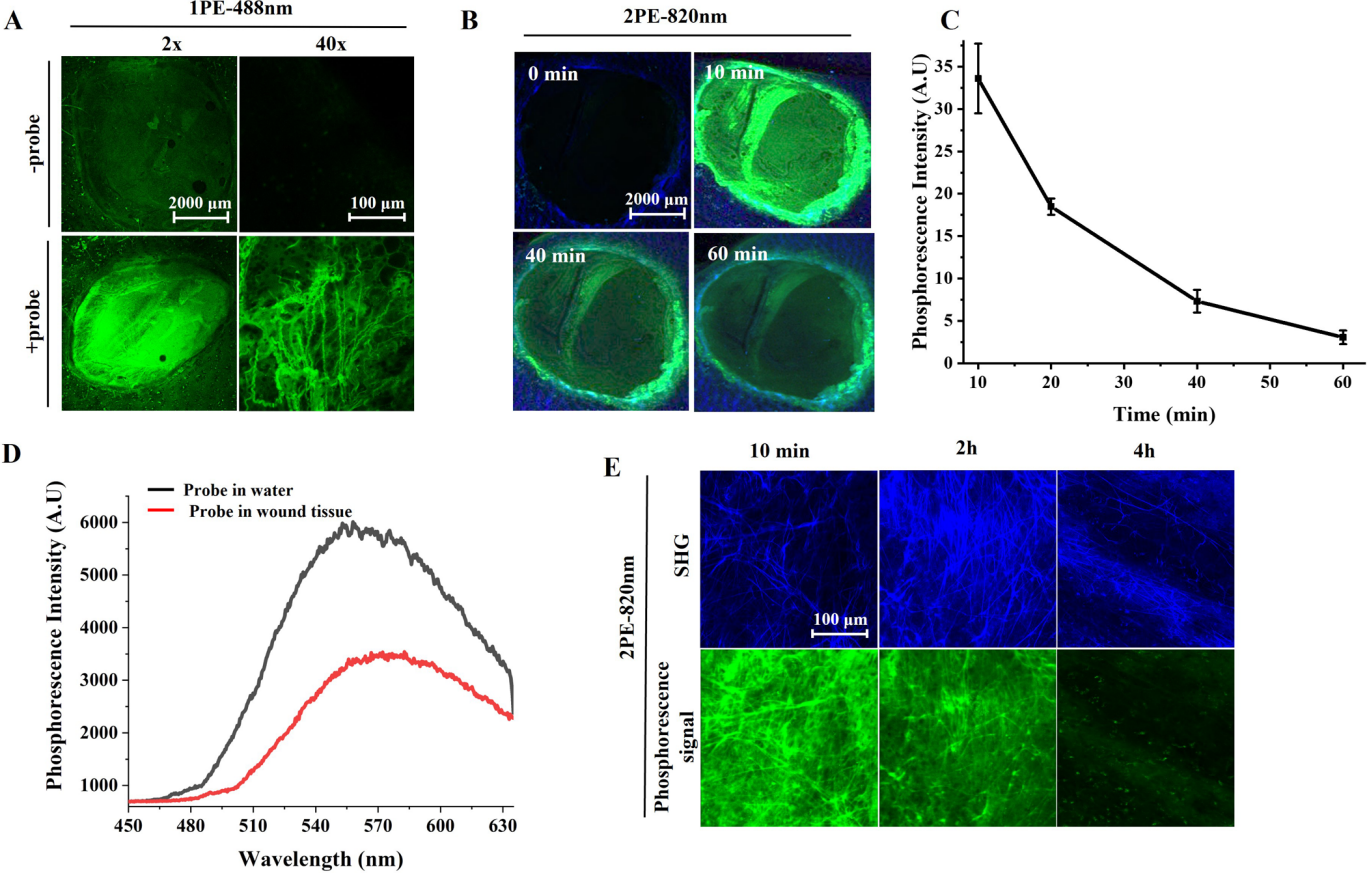
Tissue penetration and collagen visualization capability of Re^I^-probe in a cutaneous wound. (A) Single-photon excitation (1PE) luminescence imaging of mouse cutaneous wounds with (+probe) or without (-probe) Re^I^-probe. Magnifications: 2× and 40 ×. λ_ex_ = 488 nm. (B) Two-photon excitation (2PE) phosphorescence imaging of administered Re^I^-probe (green color) and second harmonic generation images of collagen (blue) at different time points postadministration. Magnifications: 2×. λ_ex_ = 820 nm. (C) Time-course phosphorescence intensities of Re^I^-probes in wounds corresponding to Figure 1B. (D) The luminescence spectrum of the Re^I^-probe in wound tissue (red line) was compared to that in water (black line), λ_ex_ = 820 nm. (E) Two-photon phosphorescence images of Re^I^-probe (green color) and second harmonic generation (SHG) images of collagen (blue color) in wound tissue. Magnifications: 40×.

By exploiting the water-soluble nature of Re^I^-probes and their collagen-binding affinity, we noninvasively administered the probe solution (20 μL, 1 mg/mL) to mouse cutaneous wounds for observation. Within a few minutes, the probe effectively penetrated the wound tissue and bound to collagen. High-magnification (40×) single-photon excited phosphorescence images revealed clear collagen morphology in the wound area (Figure 1A). To investigate the delivery kinetics and phosphorescence performance of the Re^I^-probe in cutaneous wounds, we utilized two-photon phosphorescence imaging, which reduced background autofluorescene and provided significantly better contrast (Figure 1B). Due to the diffusion and clearance of the Re^I^-probe in tissues, the phosphorescence intensity gradually decayed over the course of 1 h after application (Figure 1B & C). To confirm that the observed signal originated from the Re^I^-probe, we first measured its luminescence spectra (Figure 1D), which matched those obtained for the Re^I^-probe in aqueous solution. We then compared the morphological patterns of two-photon phosphorescence images (green in Figure 1E) with those of collagen fibers revealed by second harmonic generation (SHG, blue in Figure 1E). The patterns were quite similar and the phosphorescence images even reveal finer structures. The collagen-bound signal persisted for over 2 h, providing sufficient time for medical examination. This binding enrichment of the probes, along with the enhanced phosphorescene intensity, makes the visualization of collagen more sensitive and straightforward compared to SHG microscopy.

To ensure the biocompatibility of the Re^I^-probe, we conducted a cytotoxicity test to evaluate the viability of human fibroblast cells (L929). After 24 h of incubation, the cells were able to internalize the probes (Figure S2A). Over a monitoring period of 3 days, no significant difference in proliferation capacity was observed compared to that in the vehicle control group (Figure S2B). Additionally, no evident cytotoxicity was detected even at high concentrations of up to 500 μM (Figure S2C).

### Phosphorescence Lifetime of the Collagen-Bound Re^I^-Probe Reveals Collagen Densities and Textures

**Figure 2:**
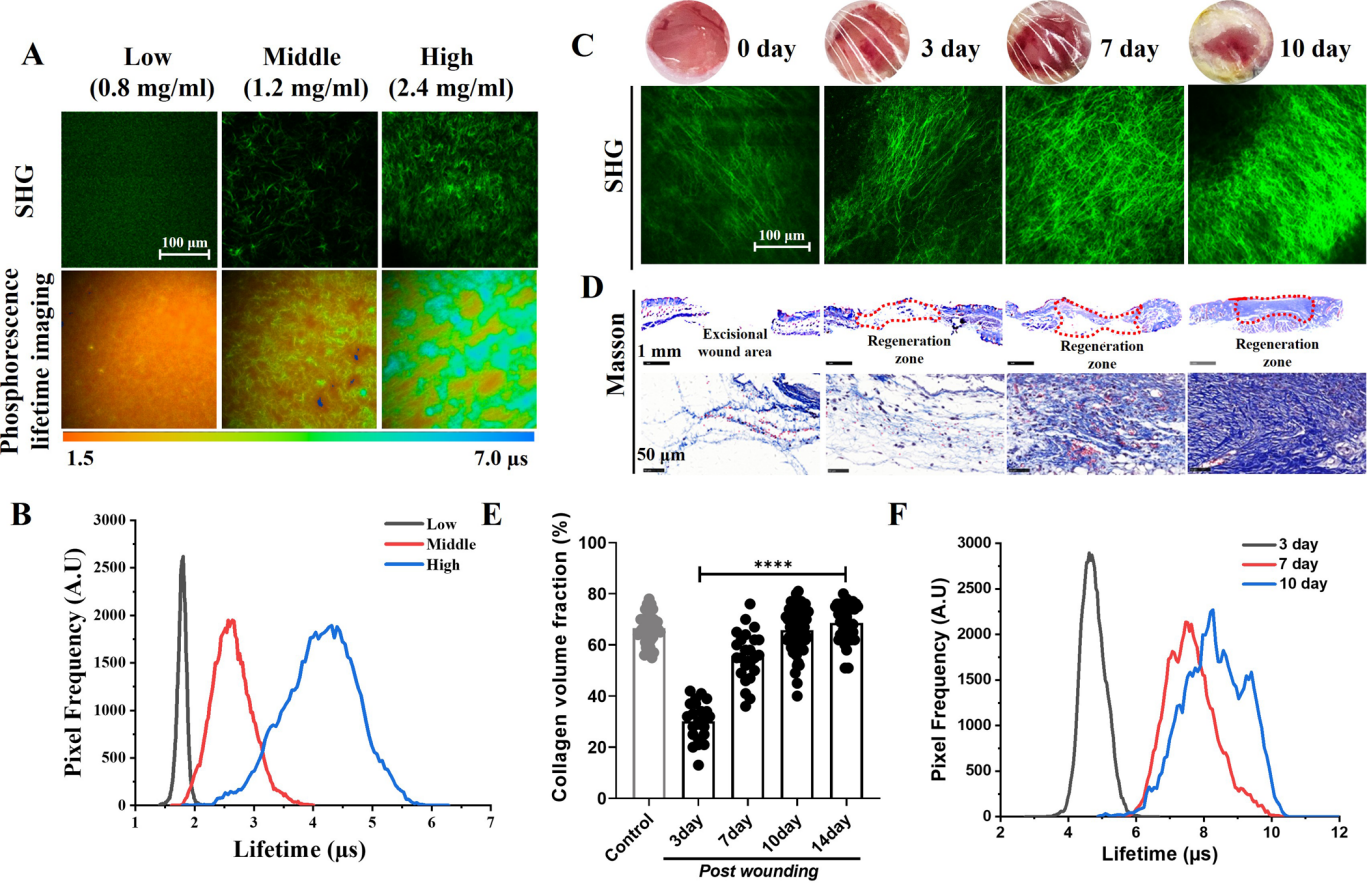
Dependence of the Re^I^-probe’s phosphorescence lifetime on collagen density. (A) The SHG (upper row) and PLIM (bottom row) images of type-I collagen gel with low, middle, and high collagen concentrations. (B) The corresponding phosphorescence lifetime histograms of Re^I^-probes in gels with different collagen densities. (C) The SHG images of wound collagen (λ_ex_ = 1100 nm) in a mouse cutaneous wound at different days postinjury. (D) Masson trichrome staining for cutaneous wounds at different days post-injury. Red dashed lines enclose the regeneration area and the subsequent row displays zoomed-in collagen staining images. (E) Quantitative analysis of collagen volume fraction based on Masson staining, referenced to Figure D. (F) The lifetime histograms of TP-PLIM in the wound area at 3, 7, and 10 days post injury.

To investigate how phosphorescence lifetime reflects collagen regeneration, we first conducted an *in vitro* test using collagen gel. We prepared gels with low, middle, and high collagen concentrations and visualized their deposition density by the 1060 nm-excitated SHG microscopy (green color in Figure 2A). As collagen density increased, the lifetime histograms from Re^I^-probe TP-PLIM images (bottom row of Figure 2A) showed a significant increase in peak value, from 1.80 to 4.31 μs (Figure 2B). The broadening of the lifetime histogram indicated greater inhomogeneity in collagen density within the field of view. We then evaluated the probe’s *in vivo* performance at different stages of cutaneous wound healing (Figure 2C). In the early stages of healing (Days 0 and 3), the average SHG intensities were higher than those observed in collagen gels, suggesting a denser collagen presence in neotissues (SHG images in Figure 2C). Collagen density increased dramatically at later stages (Figure 2D, E), leading to an increase in peak phosphorescence lifetime from 4.62 μs on day 3 to 8.23 μs on day 10 (Figure 2F). Notably, the lifetime distribution broadened from 4–6 μs to 6–10 μs and deviated from a normal distribution in the later stages of healing. These histogram features suggest heterogeneous tissue structures, which are characteristics of healed wounds.^27^ Overall, these results demonstrate that the lifetime histograms from Re^I^-probe TP-PLIM images can effectively capture collagen’s representative density, distribution inhomogeneity, and structural heterogeneity within live tissues.

### Phosphorescence Lifetime of Interstitial Re^I^-Probes Reflect Tissue Oxygenation

**Figure 3:**
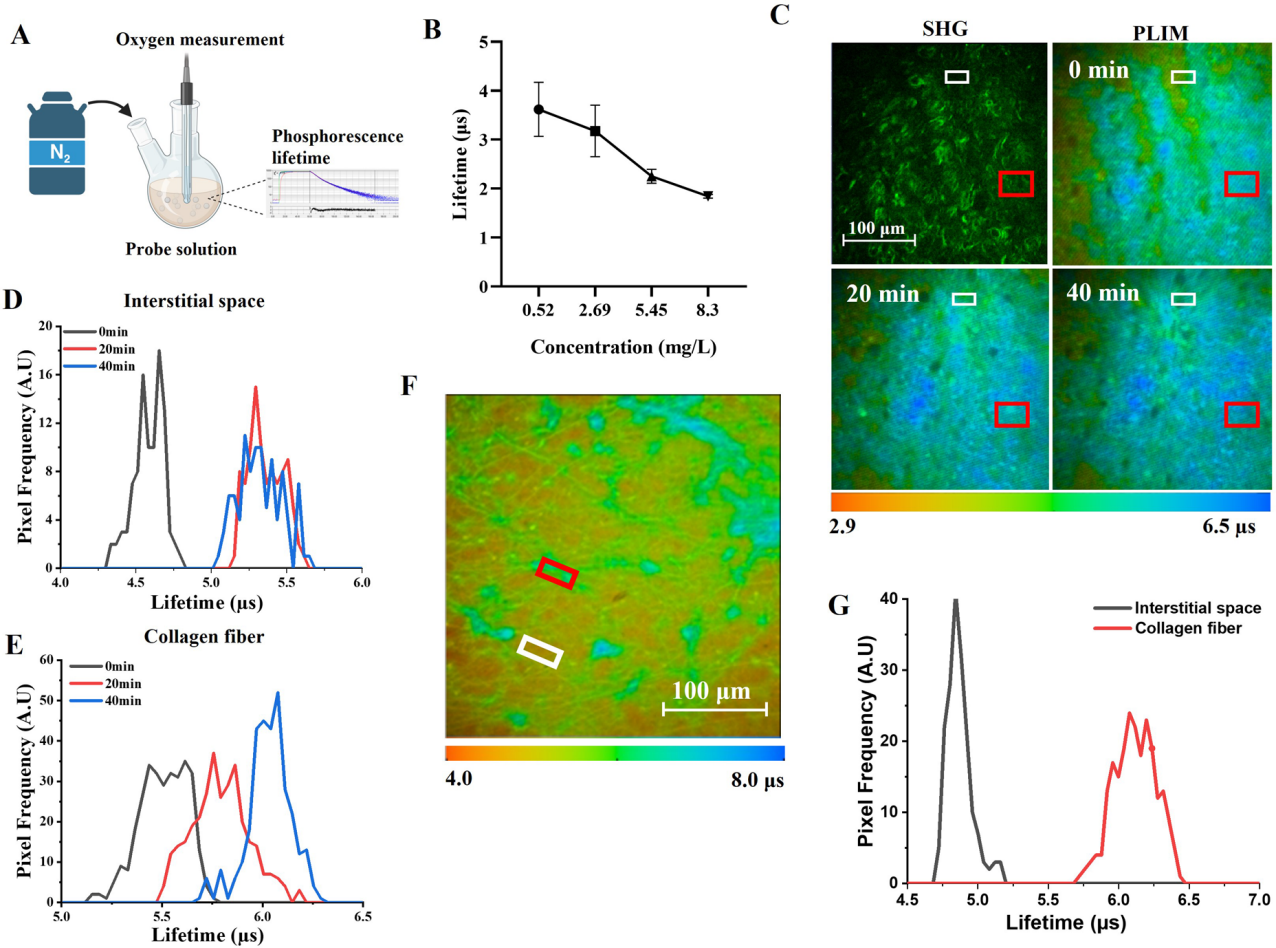
Phosphorescence lifetime of Re^I^-probe under various oxygenation conditions. (A) The experimental setup for the oxygen-deprived probe solution. (B) The lifetime of the probe solution under various O_2_ concentrations. (C) The SHG and TP-PLIM images of neotissues in a wound after continuous exposure to nitrogen gas for 0, 20, and 40 min. White and red rectangles indicate the regions of interstitial space and collagen fiber, respectively. The lifetime histograms of TP-PLIM in the (D) interstitial space and (E) collagen region after 0, 20, and 40 min of nitrogen purge. (F) The *in vivo* two-photon PLIM image of the Re^I^-probe in the cutaneous wound of a mouse. (G) The corresponding histogram of phosphorescence lifetime in collagen fiber (red rectangle in 3F) and interstitial space (white rectangle in 3F).

The Re^I^-probe’s phosphorescence lifetime, in response to changes in oxygen concentration, was initially confirmed through *in vitro* experiments. By purging N_2_ gas onto the probe solution (Figure 3A), we gradually decreased the dissolved oxygen concentration (DO), resulting in an increase in phosphorescence lifetimes from 1.84 μs (DO = 8.3 mg/L) to 3.62 μs (DO = 0.52 mg/L) (Figure 3B). In the interstitial regions, where fluid could permeate through tissues (low-SHG region marked by the white rectangle in Figure 3C), the high-viscosity environment stabilized the administrated Re^I^-probes, leading to an increase in the oxygenated phosphorescence lifetime to above 4 μs (black histogram in Figure 3D). We then purged nitrogen gas onto the same neotissue (Day 3) to create hypoxic condition. The peak phosphorescence lifetime increased up to 5.25 μs (red histogram in Figure 3D). A similar trend was observed for collagen-bound probes in the neotissue (high-SHG region marked by the red rectangle in Figure 3C), showing a peak phosphorescence lifetime of 6.12 μs under hypoxia (Figure 3E). Since the lifetime distributions in the interstitial space and collagen regions were clearly separated and distinguishable (Figure 3F & G), changes in phosphorescence lifetime within the interstitial space could specifically reflect variations in tissue oxygenation. Moreover, the dependence of collagen-bound lifetimes on the tissue oxygenation could be calibrated using measurements from the interstitial regions. That could decouple the effect of oxygenation on the phosphorescence lifetime of collagen-bound Re^I^-probes.

Unlike traditional methods that incorporate oxygen-sensitive fluorophores and phosphorescent molecules into bandages, dressings, or foils,^16,28−30^ our innovative Re^I^-probe offers a more effective and less invasive solution. Conventional approaches, while stabilizing the probes within the wound environment, often disturb the wound during application and removal, and suffer from issues like energy transfer within the host material^30^ and light scattering caused by air bubbles.^16^ In contrast, our Re^I^-probes are uniquely modified with water-soluble phosphine ligands and collagen-targeting sulfonic groups,^26^ allowing them to penetrate vessel walls and diffuse into dermal tissues effortlessly. The hydrophilic nature of these probes ensures that they can reach deep tissue areas, while the sulfonic anions enable precise targeting and stabilization on tissue collagens. This innovative design facilitates *in situ* measurement of deep tissue oxygenation, providing a more accurate and comprehensive assessment of wound healing, far beyond superficial oxygen levels.

### Re^I^-Probe Based PLIM System Can Assess the Progress of Wound Healing

Based on this dual-functional evaluation method, we were able to identify delayed healing in diabetic wounds. We selected transgenic ob/ob mice to develop animal models with chronic diabetic wounds (Figure S3A-C). The wound healing processes in ob/ob mice were significantly delayed compared to the control group (Figure S3D). Histological examination further confirmed hindered collagen regeneration and microvascular reconstruction (Figure S3E-F). After applying the probe solution locally to the wounds, we found that both groups showed increased lifetimes with the progress of wound healing (Figure S4). Averaged across the entire wound area, the mean phosphorescence lifetime in wild type mice elevated to 4.8 μs on day 3, whereas in diabetic mice, it did not exceed 4.5 μs even by day 7 (Figure S4B). Because the large field-of-view TP-PLIM used a 2× objective with a 0.1 NA, phosphorescence signals were excited and collected within a 10 μm spot diameter. Both collagen-bound and interstitial Re^I^-probes contributed to the lifetime statistics, and the spatially averaged lifetimes were typically lower than those of collagen-bound probes. To exclude pixels affected by the interstitial space, we set a gating threshold at 5.6 μs, corresponding to the upper bound of interstitial lifetimes in the histogram (Figure 3D, dashed line in Figure S4B). The proportion of pixels with lifetimes exceeding 5.6 μs was then used as an index to evaluate collagen regeneration (Figure S4C).

**Figure 4:**
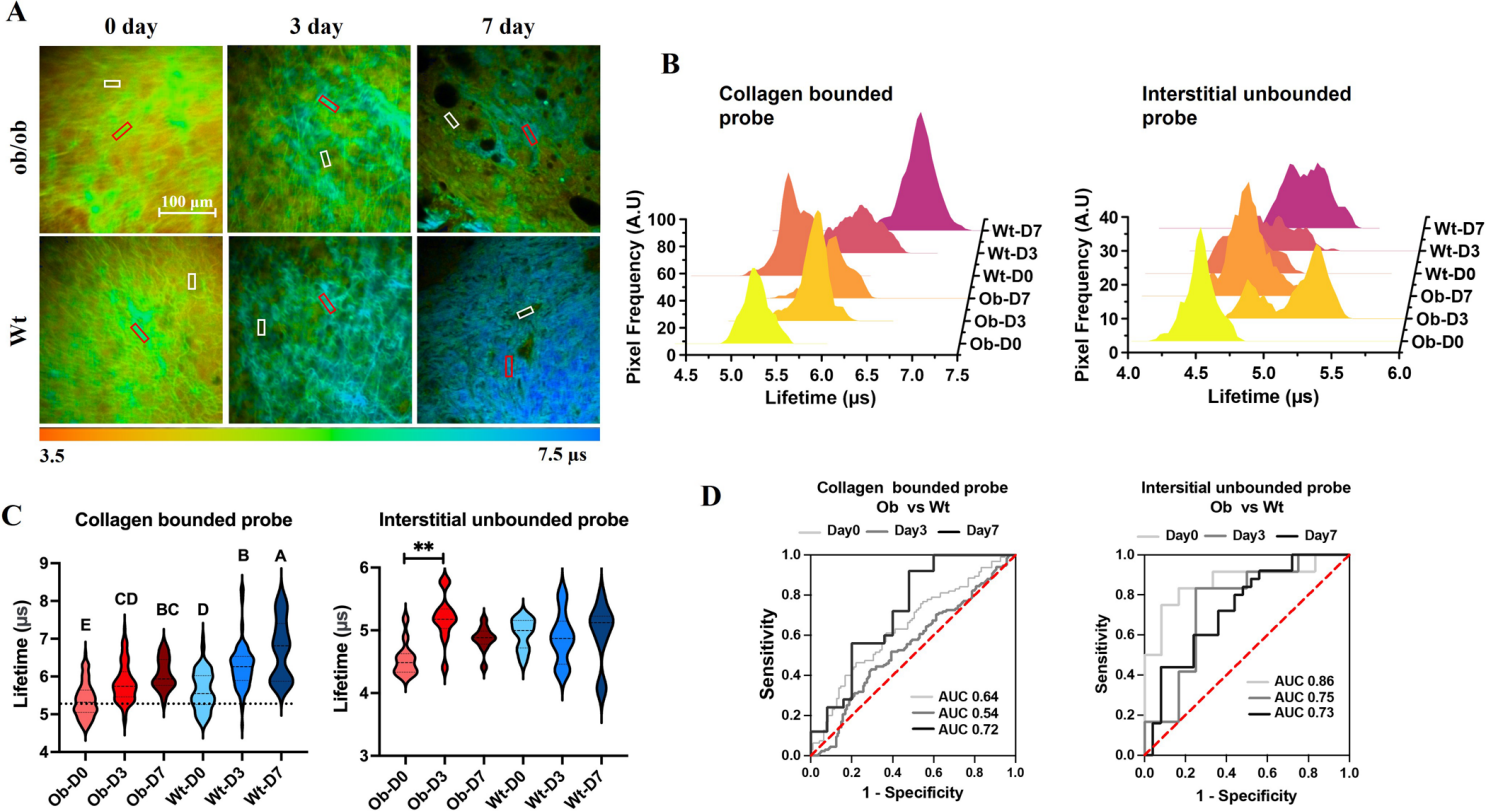
Re^I^-probe combined with PLIM technique in determining the repair process of chronic diabetic wounds. (A) The PLIM images of mouse wounds after topical Re^I^-probe application and diabetic wounds (ob/ob) at different healing times were compared with wild-type (Wt) mice. Red and white squares represent collagen area and interstitial space used for lifetime calculation, respectively. (B) The representative phosphorescence lifetime histogram of the probe in collagen-bound and interstitial unbound states. (C) The statistical comparison of lifetimes in diabetic wounds and wild-type wounds; different letters above the bars indicate statistically significant differences at *P* < 0.01 (one-way ANOVA, *N* = 6). (D) The ROC curves of diagnosing delayed healing (diabetic wounds) by collagen-bound and interstitial unbound phosphorescence lifetimes at days 0, 3, and 7 postinjury.

For a detailed analysis of wound tissues, we used a 40× objective (Figure 4A). Submicrometer resolution of the lifetime images allowed for clear separation of lifetimes associated with collagen fibers (regions in red rectangles) from those in the interstitial space (regions in white rectangles). The narrower histograms of collagen-bound phosphorescence lifetimes clearly reflected the trend of increasing lifetimes during collagen regeneration (Figure 4B). In the control group, the mean lifetime increased significantly from 5.6 μs on day 0 to 6.7 μs on day 7, indicating collagen regeneration in normal wounds. In contrast, the diabetic wounds showed a delayed increase in mean lifetime from 5.4 μs on day 0 to 6 μs on day 7 (Figure 4C). This might be due to the persistent inflammation and biofilm formation in chronic wounds, fostering a collagenolytic environment that hiders healing and predisposes to recurrence.^8^ which was consistent with histopathological evidence (Figure S3E). These results validate that changes in the phosphorescence lifetimes of collagen-bound Re^I^-probes can serve as effective indicators of collagen regeneration during wound healing, underscoring the value of a label-free, noninvasive technique for real-time monitoring of collagen dynamics.

To assess tissue oxygenation, we analyzed changes in the phosphorescence lifetime of unbound probes. Guided by the TP-PLIM image, we excluded collagen areas and focused on the interstitial spaces. At the early stages of injury, ob/ob mice showed a significant increase in phosphorescence lifetime in the interstitial space, from around 4.5 μs on day 0 to 5.2 μs on day 3 post injury, confirming the presence of a pronounced and persistent hypoxic condition (Figure 4C). This heightened hypoxia could be due to reduced blood flow, impaired angiogenesis, and disrupted oxygen transport. In contrast, during the same period, no significant change was observed in the phosphorescence lifetime of the interstitial space in control wounds during this period (4.94 to 4.88 μs). This difference may select a balance between hypoxia and oxygen supply from microcirculation regeneration in control wounds, whereas the ob/ob wounds remained in a state of persistent hypoxia. This finding underscores the utility of our approach in early differentiating wound healing quality based on oxygenation levels. By day 7 post-injury, the interstitial space lifetime in diabetic wounds had decreased, possibly due to improved blood supply and tissue oxygenation. In diabetic wounds, both the increase in collagen density and the improvement in microcirculation were significantly delayed.

In our subsequent investigation, we explored the potential of using the probe’s phosphorescence lifetime to diagnose delayed wound healing. Receiver Operating Characteristic (ROC) curves showed that the phosphorescence lifetime within the collagen-binding region could effectively differentiate wound types at the intermediate stage (day 7) with an Area Under the Curve (AUC) value of 0.72. Conversely, the phosphorescence lifetime within the interstitial region was more accurate at distinguishing wound types during the early healing stage (day 3), with an AUC value of 0.75. Additionally, we assessed the biosafety of the probe material by comparing 14-day wound healing in normal and diabetic wounds, both with and without the probe. The probe did not negatively affect the healing process (Figure S5).

### Optimized Portable FD-PLM System for Chronic Wound Assessment

**Figure 5:**
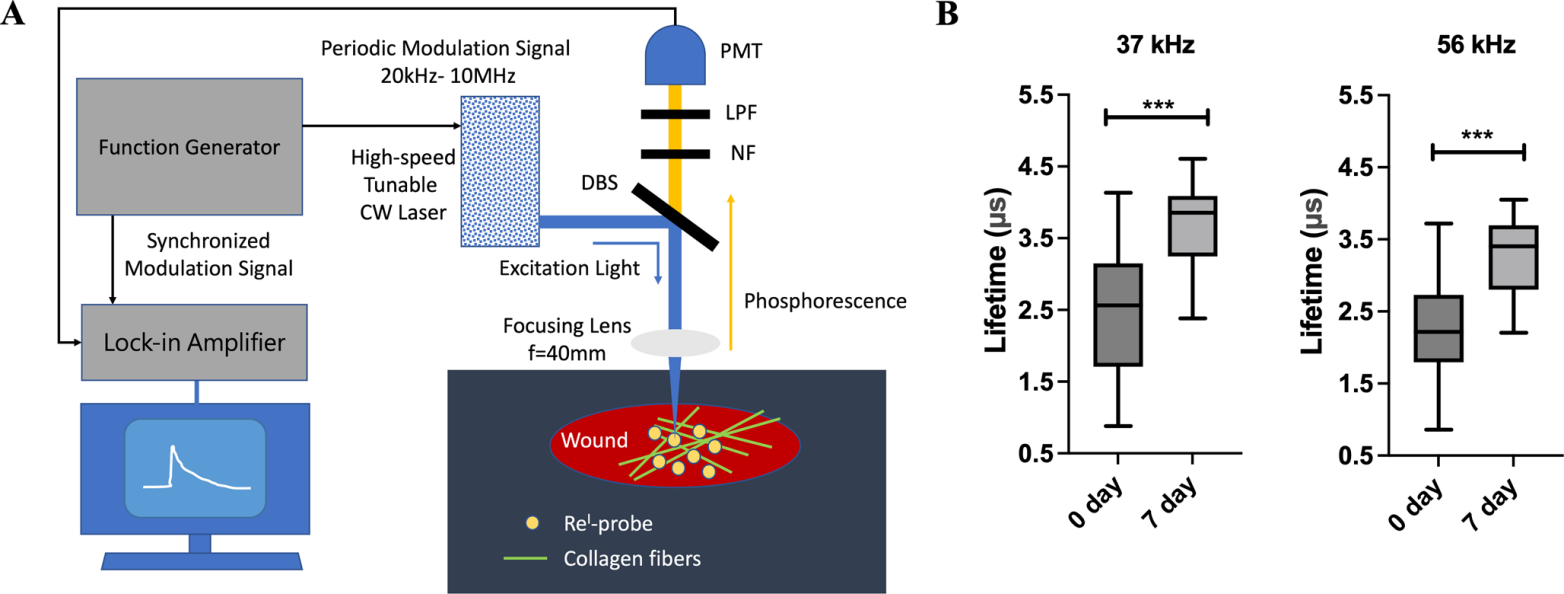
Frequency-domain phosphorescence lifetime measurement (FD-PLM) system, and the FD-PLM of Re^I^-probe in the wound environment. (A) Schematic diagram of the FD-PLM system. CW: continuous wave, LIA: lock-in amplifier, DBS: dichroic beam splitter, PMT: photomultiplier tube, NF: notch filter, LPF: long pass filter. (B) The box plots depict the lifetime measurements of Re^I^-probe at days 0 and 7 post injury. The measurements were performed at two modulation frequencies of 37 kHz and 56 kHz. (***) indicates a p-value less than 0.001 based on the two-tailed Student’s *t* test.

To enhance the portability and cost-effectiveness of wound healing assessment, we developed a frequency-domain phosphorescence lifetime measurement (FD-PLM) system (Figure 5A). A function generator provides a modulated signal output ranging from 20 kHz to 10 MHz. This modulated signal is evenly distributed by a power splitter to two output ports: one for modulating the 405 nm continuous wave (CW) laser and the other for serving as an external reference for the lock-in amplifier (LIA). The laser excitation beam is focused onto the wound area by a lens with a 4 cm focal distance, creating a focal spot diameter of 17 μm. The phosphorescence signal from the Re^I^-probe is collected by a photomultiplier tube (PMT) and digitally sampled by the LIA, allowing us to measure the relative phase shift ϕ of the phosphorescent signal with respect to the reference signal. This phase shift is directly related to the probe’s phosphorescence lifetime, providing valuable information on the progression of wound healing. To calibrate for phase shifts introduced by electronic and optical components, we used Hoechst Blue with a known fluorescence lifetime (*τ*_*ref*_)^31^ and calculated the phosphorescence lifetime using the following equation:^32^
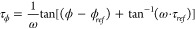
1Here, *τ*_*ϕ*_ is the phosphorescnece lifetime of the Re^I^ -probe, ω is the modulation frequency, and *ϕ*_*ref*_ is the phase shift of the Hoechst Blue fluorescence signal relative to the reference signal.

To verify the functionality of this system, we measured the phosphorescence lifetimes of the calibration phosphor Re^I^-cal (Scheme S2 in the Supporting Information) and Re^I^-probe (Scheme S1 in the Supporting Information) in solutions. As shown in Table 1, the FD-PLM measurements were consistent with the time-domain PLM (TD-PLM) results.

**Table 1:** Phosphorescence Lifetime of Re^I^-cal and Re^I^-probe in Water Solutions

	Calibration phosphor	Re^I^-probe
TD-PLM	0.4 μs	2.8 μs
FD-PLM	0.379 μs (@300 kHz)	2.704 μs (@60 kHz)

To further confirm the clinical applicability of this FD-PLM system, we examined the phosphorescence lifetime of Re^I^-probes in wounds on day 0 and on the seventh-day post-injury. We found the lifetime on the seventh-day postinjury was significantly longer than that on day 0 (Figure 5B). This increase is attributed to wound collagen regeneration and the subsequent stable binding of the Re^I^-probe to collagen, which significantly enhances its phosphorescence lifetime and quantum yield. By selecting an appropriate excitation wavelength and utilizing single-photon phosphorescence confocal microscopy, clinicians can easily visualize collagen deposition density and morphological details on various imaging scales.

Our system, even under single-point excitation, can accurately pinpoint collagen locations and determine dynamic changes in collagen density based on the decay time of phosphorescence. These findings underscore the effectiveness of our portable single-photon FD-PLM system in detecting deep-tissue collagen regeneration without the need for SHG microscopy. This innovative approach demonstrates significant potential for bedside wound healing evaluation, providing a noninvasive and precise method to monitor tissue repair.

## Conclusion

Wound healing is a complex biological process, with the oxygen level and collagen deposition serving as crucial parameters. Solely measuring oxygenation may not provide a comprehensive evaluation of wound healing. Research indicates that an adequate oxygen supply enhances collagen deposition and accelerates wound closure, highlighting the benefits of hyperbaric oxygen therapy for managing chronic diabetic wounds.^33,34^ In recent years, single indicators with dual-sensing capabilities have gained a lot of interest.^35^ Our innovative Re^I^-probe can bind to collagen within the wound tissue and distribute evenly in the interstitial space. By measuring the phosphorescence lifetime of probes in these distinct states, we can independently ascertain both the collagen density and tissue oxygen content. Our findings validate the presence of sustained hypoxia during the initial phase of diabetic wounds, leading to a consistent decline in collagen density.^36^ Therefore, the simultaneous assessment of both oxygenation and collagen regeneration offers a more comprehensive evaluation of wound healing progression and treatment efficacy.

**Figure 6:**
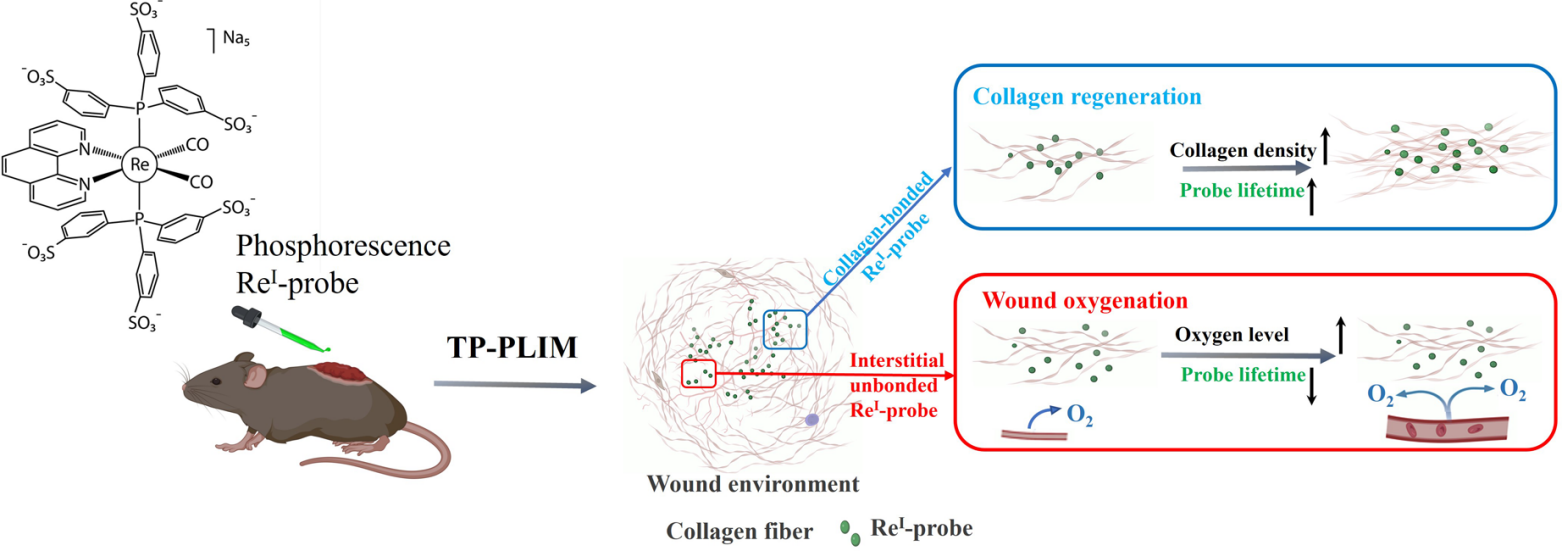
Innovative phosphorescent Re^I^-probe, when noninvasively applied to cutaneous wounds and integrated with TP-PLIM technology, allows for the detection of lifetime variations within deep wound tissue. These variations correlate with collagen regeneration and oxygenation, providing crucial information for assessing wound healing progression.

Overall, this study demonstrates the effectiveness of the Re^I^-probe, combined with deep-tissue TP-PLIM, for simultaneously monitoring collagen regeneration and tissue oxygenation during wound healing. The noninvasive application of the probe solution to the wound bed ensures effective penetration and binding to collagen. Once bound, the probe remains localized, acting as a reliable sensor for collagen density. Meanwhile, unbound probes in the interstitial space exhibit changes in phosphorescence lifetimes in response to variations in oxygen concentration. Notably, this study integrates the measurement of oxygen levels and collagen content within a single technical framework—an innovative achievement (as illustrated in Figure 6). This user-friendly approach enables consistent wound monitoring through repeated probe applications and follow-up measurement.

## Materials and Methods

### Multiphoton Luminescence and PLIM Imaging System

Multiphoton luminescence imaging was conducted using a commercial laser-scanning microscope (A1MP+Eclipse Ti-2E, Nikon instrument Inc., Japan) equipped with a fluorescence/phosphorescence lifetime imaging system (PLIM, SPC-150, Becker & Hickl). An 820 nm excitation beam was focused on tissue samples using either a 2× NA = 0.1 or a 40× NA = 1.15 water immersion objective. For in vivo study, the average power was maintained below 30 mW. To measure phosphorescence decay, the laser excitation was activated for 20 μs at a 9-kHz gating rate by the DGG-210 Module. This setup resulted in a 52 μs pixel dwell time for PLIM. During PLIM imaging, the frame time was 16.3 s, and the overall image acquisition time was 3 min per area to collect sufficient photon counts (>100 photons) for accurate curve fitting. Data analysis was performed using SPCImage 8.4 software (Becker & Hickl).

### Phosphorescence Lifetime of the Probe in Collagen Gel

To investigate the collagen-binding specificity and phosphorescence quenching rates of the Re^I^-probe with varying collagen densities, we measured the TP-PLIM of the Re^I^-probe administered to collagen gels in vitro. The collagen gel was prepared by mixing three components (A: rat collagen I; B: 10X Ham’s F-12; C: recombinant buffer solution containing 2.2 g NaHCO_3_ in 100 mL of 0.05N NaOH, and 200 mM HEPES) in an 8:1:1 ratio. The solution was then incubated overnight in a humidified incubator at 37 °C with 5% CO_2_ to form collagen fibers. By adjusting the volume of collagen I added, we prepared three collagen gels with different densities: low concentration gel (0.8 mg/mL), medium concentration gel (1.2 mg/mL) and high concentration gel (2.4 mg/mL). Subsequently, the collagen gels were treated with Re^I^ probe solution (1 mg/mL, 500 μL) for 10 min. After that, the probe solution was removed, and the performance of the Re^I^-probe was observed using a PLIM platform. The SHG signals of collagen was obtained at excitation wavelength of 1060 nm, and the phosphorescence signals of the probe were tracked at an excitation wavelength of 820 nm. Finally, the lifetime histogram of each PLIM image was generated, and the peak lifetime was chosen as the representative value shown in figures.

### Phosphorescence Lifetime of the Probe in Solution with Different Oxygen Concentrations

To assess the phosphorescence quenching rates of the Re^I^-probe in free states, we measured the TP-PLIM of the probe in solution with different oxygen concentrations. The probe sample was dissolved in PBS and was placed on a bottom-glass Petri dish for 820 nm excited TP-PLIM. The dissolved oxygen concentration in the measurement chamber was adjusted by an O_2_–N_2_ gassing system and monitored by an O_2_ meter. The phosphorescence decay traces under different oxygen concentrations were measured, and the lifetime was recorded after fitting.

### Retention of Re^I^-Probe in Wounds

For the whole wound scanning, a 2× NA = 0.1 objective lens was used, and the phosphorescence signals of the probe were captured after implantation at different time points (0, 10, 40, and 60 min). ImageJ was used to perform the intensity calculation. For depth-dependent examinations, a 40× NA = 1.15 water immersion objective was used at different time points (10 min, 2 h, and 4 h). Both the phosphorescence signals of the probe and the SHG signals of collagen were recorded. During the experiments, anesthesia was achieved by injecting Avertin intraperitoneally with an appropriate dosage.

### Mouse Excisional Wound Model

All animal experiments adhered to a protocol sanctioned by the University of Macau’s Subpanel on Animal Research Ethics (UMARE-019-2022). Male nude mice, aged 6–8 weeks and weighing between 20 and 23 g, were sourced from the University of Macau’s animal facility. The excisional wound model was employed as delineated.^37^ In summary, each mouse was anesthetized before two full-thickness skin wounds, each 5 mm in diameter, were inflicted with a biopsy punch on either side of the midline. A silicone splint encircled each wound to maintain its position, and a 3M Tegaderm dressing provided coverage. The wounds were imaged intravitally by TP-PLIM at specified intervals postinjury (e.g., days 0, 3, 7, and 10).

### Development of Diabetic Chronic Wound Model

For the purpose of conducting experiments on diabetic chronic wounds, transgenic mice (B6.Cg-Lep^ob^/J) were utilized. Following mating and breeding, hybrid Lep^ob^ mice were identified. The ob+ mice served as the control group, representing normal physiological conditions, while the ob/ob mice were considered diabetic. The skin wound model was implemented on both types of mice 12 weeks after breeding. After hair removal from the dorsal surface and anesthesia, two full-thickness skin wounds were created as previously described to compare the wound healing rate with wild-type mice. Photographs of the wound area were taken at days 0, 3, 7, 10, and 14 post-wounding. The wound margin was marked on images, and the wound area was calculated with an image analysis program (www.getpaint.net). The investigators measuring the wounds were blinded to the treatment groups. The percentage of wound closure was calculated as follows: (area of original wound – area of current wound)/(area of original wound) × 100%. Methods for comparing wound healing quality in diabetic and wild-type wounds reference previous work.^37^

### Measuring Phosphorescence Lifetime of the Probe in Mouse Wounds

For the preparation of the probe solution, the Re^I^-probe was dissolved in sterilized PBS (1 mg/mL) and then passed through a syringe filter with a 0.22 μm pore size. A 20 μL probe solution was topically applied to cutaneous wounds of mice. After 10 min of incubation, the residual solution was removed. The wound was then covered with a cover glass and intravitally observed using a Nikon Inverted Multiphoton Microscope Eclipse equipped with a phosphorescence lifetime imaging system.

### Ex Vivo Calibration of Phosphorescence Lifetime of Probes in Wound Skin Tissue

The fresh skin wound was excised from a sacrificed mouse and then soaked in Re^I^-probe (1 mg/mL) at 37 °C. After 10 min of incubation, the residual solution was removed, and the sample was bathed in PBS. The prepared wound sample in PBS was then placed on a bottom-glass Petri dish and observed with a PLIM system under 20% oxygen ambient condition (0 min) and depleted oxygen with a nitrogen purge (20 and 40 min). The collagen regions and interstitial space in the wound tissue were selected respectively, and the phosphorescence decay traces under two extreme oxygen levels were measured. The lifetime was calculated after fitting.

### Masson Trichrome Staining

Mice were sacrificed at designated time points, and skin samples, including the wound and 2 mm of the adjacent skin, were dissected by using an 8 mm biopsy punch. Tissue specimens were fixed in 4% paraformaldehyde, dehydrated in a graded ethanol series, and embedded in paraffin. Sections with a 5 μm thickness were stained with a Masson trichrome staining kit according to the manual (Heart Biological Technology). The blue area was defined as positive staining for collagen, and the collagen volume fraction was defined as the amount of the total collagen area in relation to the total area of the biopsy.

### FD-PLM System

In the FD-PLM system, a function generator (33600A, Keysight) was utilized to generate a periodic sinusoidal modulation signal with a frequency range of 20 kHz to 10 MHz, an amplitude of 1.0 Vpp, and an offset of 1.5 V. This signal was split to provide both the excitation modulation for the high-speed tunable continuous wave (CW) laser (MDL-NS-405-25mW, Changchun New Industries Optoelectronics Technology Co., Ltd.) and the synchronized modulation signal for the lock-in amplifier (LIA, SRA44, Stanford Research Systems).

The CW laser, modulated by the function generator, served as the excitation light source, directing the beam toward the wound site to induce phosphorescence. The DBS (FF485-Di03, Semrock) splits the phosphorescent signal from the reflected excitation light. After focusing excitation by a lens with a 4 cm focal distance, the epi-collected phosphorescent light then passed through the DBS, a notch filter (NF; NF405-13, Thorlabs), and a long pass filter (FEL0450, Thorlabs) before being detected by the photomultiplier tube (PMT; H10721-20, HAMAMATSU). The PMT was powered by a regulated power supply (C10709, HAMAMATSU) set at 0.6 V. The electrical current output from the PMT was subsequently converted to a voltage signal by a transimpedance amplifier (C6438-01, HAMAMATSU), which was then relayed to the LIA for the precise analysis and quantification of the phosphorescence lifetime signals.

### Statistical Analysis

Data are presented as mean ± SD. GraphPad Prism 10.0 software was used for statistical analysis. An unpaired Student’s *t* test and one-way ANOVA with Tukey’s post hoc test were performed to determine the significant differences between two groups and among three groups, respectively. A p-value of <0.05 was regarded as statistically significant.

## Data Availability

All raw and processed data can be provided upon request.

## Supporting Information Available

Additional Materials and Methods section; Scheme S1, synthesis of Re^I^-probe; Scheme S2, synthesis of Re^I^-cal; Figure S1, ^31^P{^1^H} NMR and ^1^H NMR of Re^I^-cal; Figure S2, biocompatibility of the Re^I^-probe; Figure S3, physiological characterization of diabetic ob/ob mice; Figure S4, TP-PLIM images and corresponding lifetime histograms of mice wounds after topical Re^I^-probe application; and Figure S5, biosafety assessment of the Re^I^-probe in wounds (PDF)
