# Supplementary material for: Assessing Wound Healing in Vivo Using a Dual-Function Phosphorescent Probe Sensitive to Tissue Oxygenation and Regenerating Collagen
Source: ACS Appl Mater Interfaces. 2024 Dec 27;17(1):398–407. doi: 10.1021/acsami.4c15069 (PMC11783361; doi:10.1021/acsami.4c15069)
Supplement: Supplementary file 1 — am4c15069_si_001.pdf [file am4c15069_si_001.pdf]

# Supporting Information

## Assessing wound healing in vivo using a dual-function phosphorescent probe sensitive to tissue oxygenation and regenerating collagen

Xiaoyan Wang[1#], Zhiming Zhang[1#], Xuhao Ye[1], Liping Chen[2], Weiming Zheng[1], Ning Zeng[3,4*], Zhouji Shen[5], Fei Guo[6], Igor O. Koshevoy[7], Kristina S. Kisel[7], Pi-Tai Chou[8]*, and Tzu-Ming Liu[1]*

[1]Institute of Translational Medicine, Faculty of Health Sciences & Ministry of Education Frontiers Science Center for Precision Oncology, University of Macau, Taipa 999078, Macau, China
[2]Department of Pediatric Surgery, Guangzhou Institute of Pediatrics, Guangdong Provincial Key Laboratory of Research in Structural Birth Defect Disease, Guangzhou Women and Children's Medical Center, Guangzhou Medical University, Guangzhou 510623, Guangdong, China
[3]First Department of Hepatobiliary Surgery, Zhujiang Hospital, Southern Medical University, Guangzhou 510280, China
[4]Guangdong Provincial Clinical and Engineering Center of Digital Medicine, Guangzhou 510280, China
[5]Ningbo Medical Center LiHuiLi Hospital, The Affiliated LiHuiLi Hospital of Ningbo University, Ningbo, Zhejiang 315040, China
[6]Ningbo Institute of Innovation for Combined Medicine and Engineering (NIIME), The Affiliated Lihuili hospital of Ningbo University, Ningbo, Zhejiang 315040, China
[7]Department of Chemistry, University of Eastern Finland; FI-70211, Joensuu, Finland
[8]Department of Chemistry, National Taiwan University, Taipei 10617, Taiwan

[#]These authors contributed equally.
*Corresponding author: Ning Zeng, chen_ning16@foxmail.com; Pi-Tai Chou, chop@ntu.edu.tw; Tzu-Ming Liu, tmliu@um.edu.mo

## Materials and Methods

### Synthesis General Comments

1,3,5-Triaza-7-phosphaadamantane (PTA), $Na_3$-tris-(3-sulfophenyl)phosphine (TPPTS), $[Re(phen)(CO)_3(NCMe)](CF_3SO_3)$ and $[Re(phen)(CO)_3(H_2O)](CF_3SO_3)$ were prepared according to published procedures. Toluene was distilled from Na-benzophenone ketyl under nitrogen prior to use. Other reagents and solvents were used as received. The solution $^1H$ and $^{31}P\{^1H\}$ spectra were recorded on a Bruker Avance 400 spectrometer. Mass spectra were measured on a Bruker APEX-Qe Qh-FT-ICR instrument in $ESI^-$ modes. Microanalyses were carried out at the analytical laboratory of the University of Eastern Finland.

### Synthesis of $Re^I$-probe

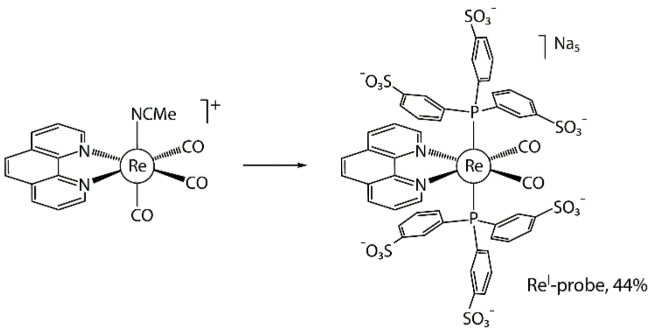


**Scheme S1**. Synthesis of Re$^I$-probe: TPPTS, water/ethanol 1:1 v/v, autoclave, 220 $^oC$, 12 h, $N_2$.

**[Re(phen)(CO)$_2$(TPPTS)$_2$]Na$_5$ (Re$^I$-probe).** [Re(phen)(CO)$_3$(CH$_3$CN)](CF$_3$SO$_3$) (118 mg, 0.184 mmol), TPPTS (262 mg, 0.461 mmol) and the mixture of water with ethanol (1:1 v/v, 15 ml) were placed in a 60 ml autoclave, pressurized with nitrogen to 25 atm and heated at 220°C overnight. The resulting yellow solution was evaporated and the residue was washed with cold methanol (2 ml). Recrystallization by a gas phase diffusion of acetone into water solution of Re$^I$-probe at room temperature to afforded bright yellow fine crystalline material (125 mg, 44%). ESI-MS (*m/z*): [M+Na$_4$]$^-$ 1512.75 (calcd 1512.85), [M–TPPTS+Na]$^-$ 944.88 (calcd 944.94). IR (CH$_3$CN, *ν*(CO), cm$^{-1}$). $^1$H NMR (D$_2$O, 298 K; δ): 8.43 (d, $J_{HH}$ 5.0 Hz, 2H, 2,9-H phen), 8.23 (d, $J_{HH}$ 8.1 Hz, 2H, 3,8-H phen), 7.83 (s, 2H, 4,7-H phen), 7.60 (m, 6H, TPPTS), 7.41 (m, 6H, TPPTS), 7.30 (m, 12H, TPPTS), 7.17 (dd, $J_{HH}$ 8.1 and 5.0 Hz, 2H, 5,6-H phen). $^{31}$P{$^1$H} NMR (D$_2$O, 298 K; δ): 24.1 (s). Anal. Calcd for $C_{50}H_{32}N_2Na_5O_{20}P_2ReS_6 \cdot 4H_2O$: C 37.33; H 2.50; N 1.74; S 11.96. Found: C 37.22; H 2.52; N 1.69; S 12.08.

**Synthesis of calibration phosphor Re$^I$-cal**

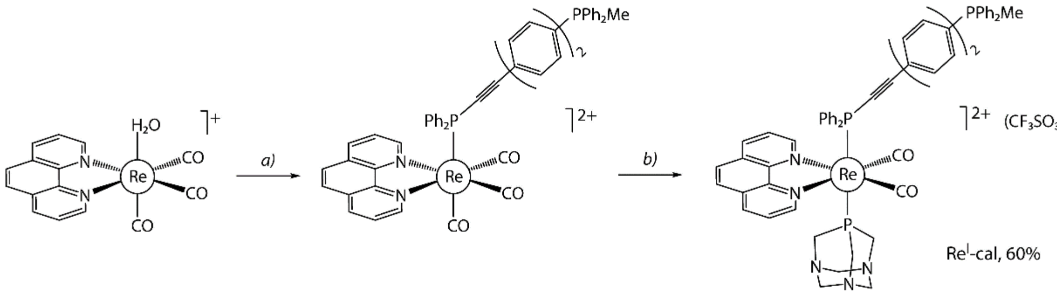


**Scheme S2.** Synthesis of Re$^I$-cal:**(A)***)* PP$^+$, toluene/propanol 1:1 v/v, reflux, 12 h, $N_2$; (B) PTA, Me$_3$NO.2H$_2$O, acetone/methanol 15:1 v/v, room temperature, 12 h, $N_2$.

**(4'-((Diphenylphosphaneyl)ethynyl)-[1,1'-biphenyl]-4-yl)(methyl)diphenylphosphonium triflate (PP$^+$).** 4-bromo-4'-ethynyl-1,1'-biphenyl (1.75 g, 6.8 mmol) was degassed in a Schlenk flask and freshly distilled tetrahydrofuran (50 ml) was added under a nitrogen atmosphere. Solution was cooled to –78 $^oC$ and treated dropwise with n-BuLi (1.6 M in hexanes, 10 ml, 16 mmol) within 20 min. The mixture turned into a pale suspension, which was stirred for 4 h at a temperature below –40 $^oC$. Then chlorodiphenyl phosphine (3.3 g, 15 mmol) was added dropwise and the resulting mixture was left to stir overnight. The reaction was quenched with methanol (5 ml), solvents were evaporated and oily residue was separated using column chromatography (Sillica gel, column ø2.5×15 cm, eluent dichloromethane:hexane 1:2 v/v mixture). Second fraction was collected and evaporated to give the product ((4'-(diphenylphosphaneyl)-

[1,1'-biphenyl]-4-yl)ethynyl)diphenylphosphane as pale amorphous solid (2.2 g, 59%). $^{31}P\{^1H\}$ NMR (202 MHz, $CDCl_3$, 298 K; *δ*): –5.5 (s, 1P, $C_6H_4$-$PPh_2$), –32.7 (s, 1P, $C_2$-$PPh_2$). $^1H$ NMR (500 MHz, $CDCl_3$, 298 K; *δ*): 7.70–7.65 (m, 4H), 7.62–7.54 (m, 6H), 7.40–7.32 (m, 18H).

The resulting diphosphine (1g, 1.83 mmol) was dissolved in freshly distilled toluene (20 ml) under a nitrogen atmosphere and cooled to –20 ºC. Then a solution of methyl triflate (305 mg, 1.86 mmol) in toluene (5 ml) was added and a mixture was allowed to reach room temperature within ca. 2 h. The mixture was concentrated to ca. 10 ml and diluted with diethyl ether (10 ml). The liquids were decanted from oily precipitate, which was washed with ether (2×10 ml) and then purified by column chromatography (Sillica gel, ø2.5×15 cm, eluent dichloromethane:methanol 25:1 v/v mixture) to give yellow powder after evaporation (1.14 g, 87 %). $^{31}P\{^1H\}$ NMR (202 MHz, $CDCl_3$, 298 K; *δ*): 22.0 (s, 1P, $C_6H_4$-$P^+MePh_2$), –32.8 (s, 1P, $C_2$-$PPh_2$). $^1H$ NMR (500 MHz, $CDCl_3$, 298 K; *δ*): 7.88–7.85 (m, 2H), 7.81–7.71 (m, 4H), 7.70–7.57 (m, 16H), 7.39–7.31 (m, 6H), 2.95 (d, $^2J_{HP}$ 13.4 Hz, 3H).

**[Re(phen)(CO)$_2$(PP$^+$)(PTA)](CF$_3$SO$_3$)$_2$ (Re$^I$-cal).** [Re(phen)(CO)$_3$($H_2O$)]($CF_3SO_3$) (200 mg, 0.32 mmol) and **PP$^+$** (250 mg, 0.35 mmol) were suspended in 1-propanol (7 ml) and toluene (7 ml), the mixture was degassed and refluxed under a nitrogen atmosphere overnight. Solvents were removed, yellow residue was dissolved in acetone (25 ml) and excess of PTA (105 mg, 0.66 mmol) was added. The mixture was degassed and treated dropwise with a solution of $Me_3NO.2H_2O$ (40 mg, 0.4 mmol) in acetone-methanol (6 ml, 2:1 v/v mixture) under a nitrogen atmosphere. Resulting yellow-orange solution was stirred overnight at room temperature, then it was evaporated and orange solid was purified by column chromatography (neutral alumina, ø1.5×7 cm, eluent dichloromethane:methanol 20:1 v/v mixture; then neutral alumina, ø1.5×7 cm, eluent acetonitrile:methanol 35:1 v/v mixture) to give complex Re$^I$-cal as orange solid (280 mg, 60%). $^{31}P\{^1H\}$ NMR (202 MHz, acetone-*d*$_6$, 298 K; *δ*): 22.7 (s, 1P, $C_6H_4$-$P^+MePh_2$), 6.3 (d, $^2J_{PP}$ 165 Hz, 1P, $C_2$-$PPh_2$), –72.1 (d, $^2J_{PP}$ 165 Hz, 1P, PTA). $^1H$ NMR (500 MHz, acetone-*d*$_6$, 298 K; *δ*): 9.17 (dq, $J_{HH}$ 5.1 and 1.3 Hz, 2H), 8.77 (ddd, $J_{HH}$ 8.2, 2.1 and 1.1 Hz, 2H), 8.25 (s, 2H), 8.14 (dd, $J_{HH}$ 8.6 and 3.0 Hz, 2H), 8.01–7.85 (m, 12H), 7.83–7.78 (m, 4H), 7.39–7.27 (m, 12H), 4.33 (d, $J_{HH}$ 12.6 Hz, 3H), 4.21 (d, $J_{HH}$ 12.6 Hz, 3H), 3.78 (s, 6 H), 3.24 (d, $^2J_{HP}$ 14.1 Hz, 3H). ESI:MS (*m/z*): $[M]^{2+}$ 570.6402 (calcd 570.6390). Anal. Calcd for $C_{61}H_{51}F_6N_5O_8P_3ReS_2$: C, 50.90; H, 3.57; N, 4.87; S, 4.46. Found: C, 51.26; H, 3.38; N, 4.45; S, 4.11.

### *In vitro* biocompatibility test

The L929 fibroblast cells were seeded into 24-well plates with the concentration of 5×10^4 cells/mL and added with either Re$^I$-probe (50 μM) or vehicle control. After 24 hours incubation, cells were washed with PBS and changed with fresh culture medium, cell proliferation rate was monitored according to the IncuCyte manufacturer's instructions. The cytotoxicity of Re$^I$-probe was assessed by cell counting kit-8 (CCK-8) cell viability assay. The L929 cells were seeded in 96-well plate with a concentration of $5\times10^4$ cells/mL, five different concentrations of Re$^I$-probe were added to each well (10-500 μM). After 24 hours of incubation, the cells were washed with PBS and then incubated in fresh medium containing the CCK-8 reagent. They were kept in the incubator for an additional 2 hours. The absorbance of each sample was measured at 450 nm. The uptake of probes in L929 cells was also validated via two-photon luminescence microscopy and spectroscopy under the illumination of 820-nm femtosecond laser pulses.

### Supplemental Figure and Legends

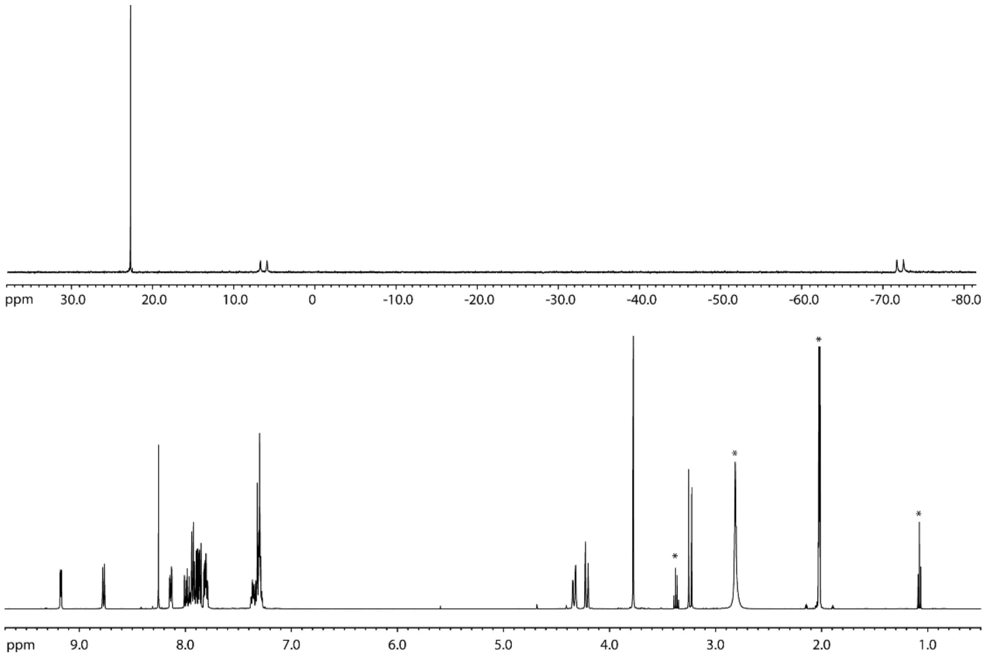


**Figure S1.** The $^{31}P\{^{1}H\}$ NMR (above) and $^{1}H$ NMR (below) of $Re^{I}$-cal (signals denoted with asterisk correspond to residual solvent peak, water and diethyl ether).

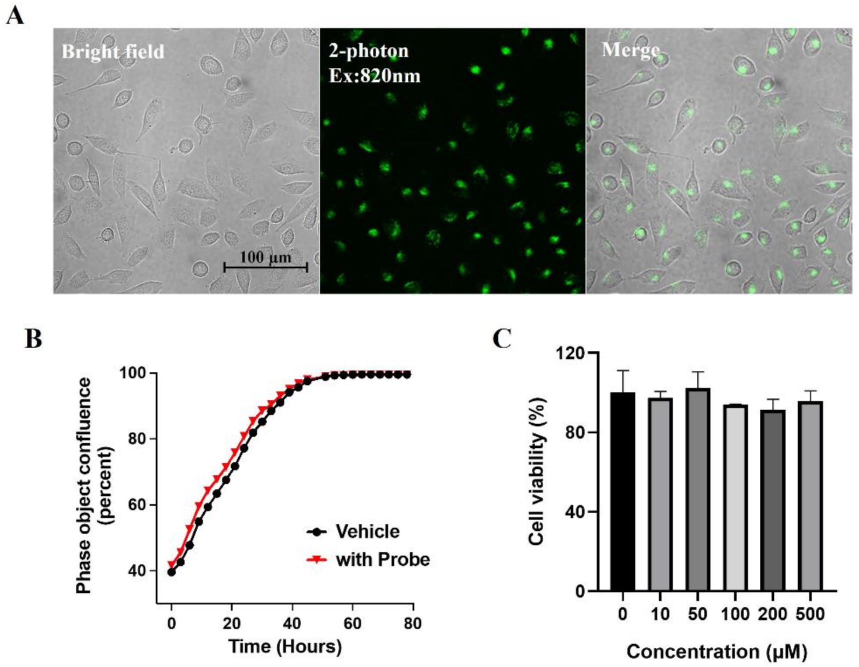


**Figure S2.** The biocompatibility of $Re^{I}$-probe. **(A)** The probe (50 μM) uptake image of L929 cells at 24 h post administration; $\lambda_{ex}$ = 820 nm. **(B)** Time-course proliferation of L929 cells with $Re^{I}$-probes (50 μM) and without Vehicle, N=3; **(C)** The viability of L929 cells treated with probe under various concentrations for 24 h.

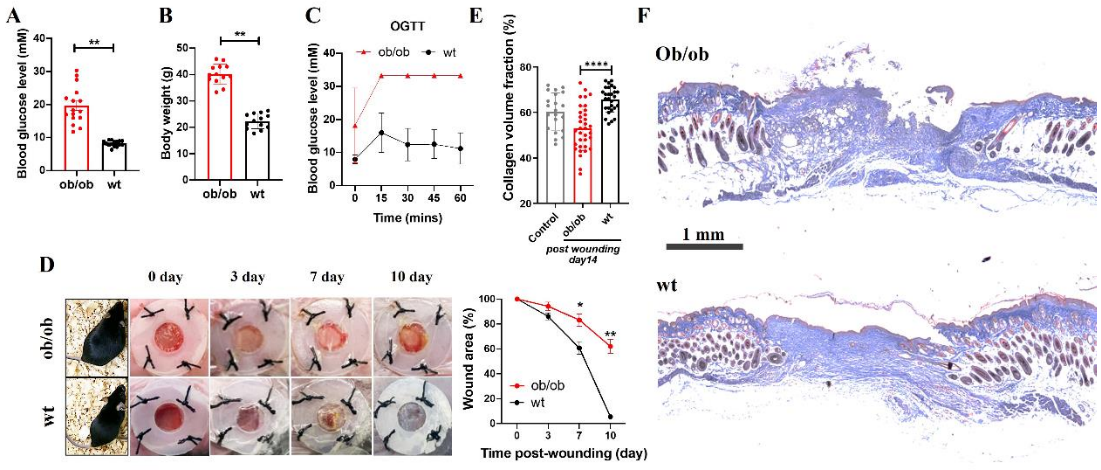


**Figure S3.** The physiological characterization of diabetic ob/ob mice. **(A-C)** Mice were assessed by **(A)** Blood glucose (N=15), **(B)** Body weight (N=13), and **(C)** blood glucose level during oral glucose tolerance test (OGTT) (N=3). **(D)** Wound recovery evaluation at various timepoints post-wounding on diabetic ob/ob mice and wild type (wt) mice (N=6). **(E)** Comparison of collagen volume fraction in the skin of control mice, day 14 post-wounding ob/ob mice and wild-type mice (N=4). **(F)** Representative collagen staining images of skin at the 10$^{th}$ day post-wounding in ob/ob and wild type mice.

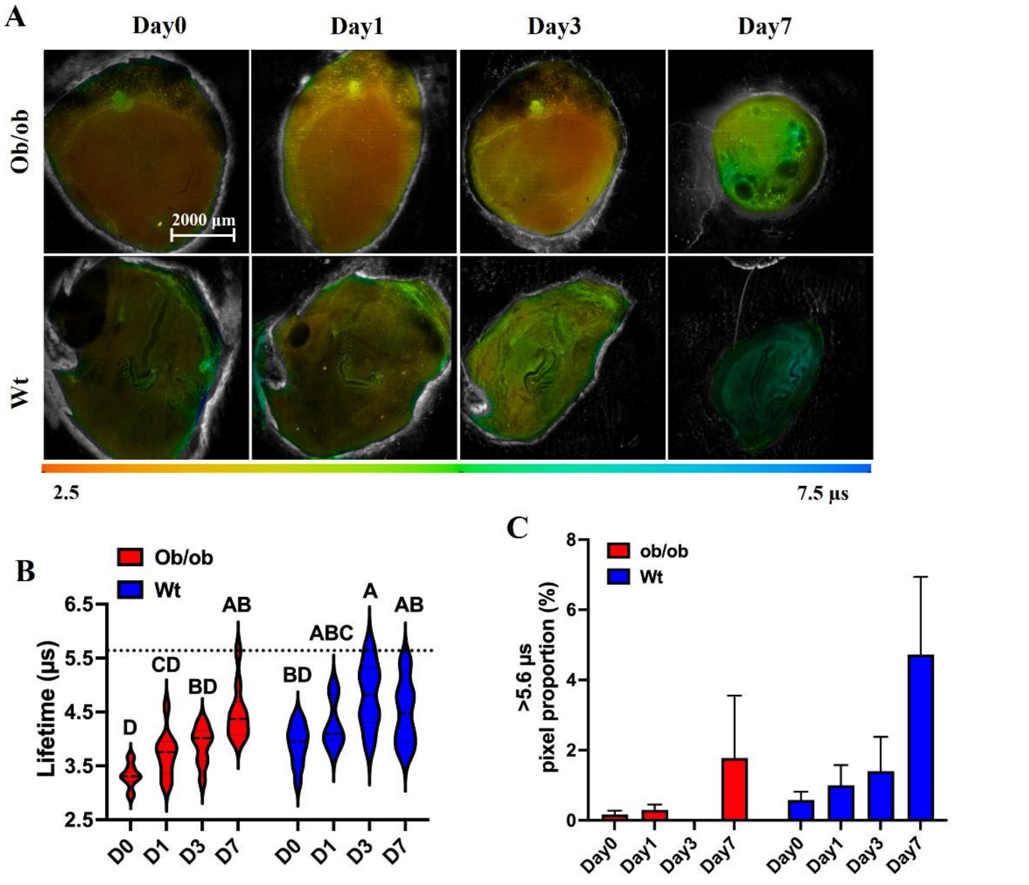


**Figure S4.** **(A)** The TP-PLIM images (objective NA = 0.1) and **(B)** corresponding lifetime histograms (violin plots) of mice wound after topical Re$^{I}$-probe application. The entire wound was analyzed as a single image. Color bars indicate a 2.5-7.5 μs range. Fields of view (6 mm × 6 mm). Different letters above the bars indicate statistically significant difference at P<0.01 (one-way ANOVA, N=6). **(C)** The proportion of pixels with lifetimes exceeding 5.6 μs in histograms of (B).

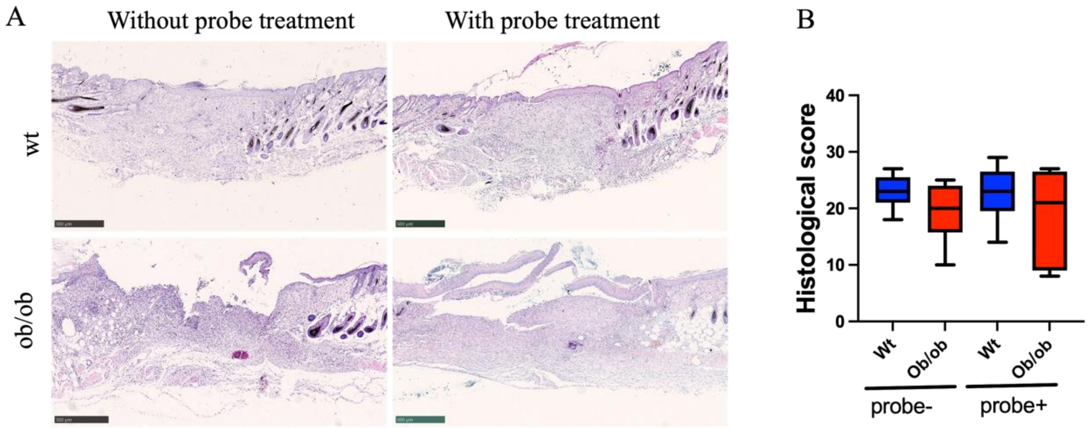


**Figure S5.** Biosafety assessment of Re$^{I}$-probe in wounds: **(A)** HE staining was performed on 14-day wounds with and without the probe material to compare healing quality (scale bar = 500 μm). **(B)** Histological evaluation of HE-stained samples. Assessed parameters include re-epithelialization, cellular infiltration, granulation tissue formation, and angiogenesis.
